# 13,14-bis(cis-3,5-dimethyl-1-piperazinyl)-β-elemene, a novel β-elemene derivative, shows potent antitumor activities via inhibition of mTOR in human breast cancer cells

**DOI:** 10.3892/ol.2013.1213

**Published:** 2013-02-27

**Authors:** XIAO-FEI DING, MAO SHEN, LI-YING XU, JIN-HUA DONG, GUANG CHEN

**Affiliations:** 1 Schools of Medicine, Taizhou College, Taizhou, Zhejiang 318000;; 2 Pharmaceutical and Chemical Engineering, Taizhou College, Taizhou, Zhejiang 318000;; 3 Key Laboratory of Structure-Based Drug Design and Discovery, Ministry of Education, Shenyang Pharmaceutical University, Shenyang, Liaoning 110016, P.R. China

**Keywords:** 13,14-bis(cis-3,5-dimethyl-1-piperazinyl)-β-elemene, β-elemene, mTOR, autophagy, cancer

## Abstract

Elemene has been approved for the treatment of advanced cancer in China, however, it inhibits cell growth only at high concentrations and is an essential oil with poor water solubility and stability. The discovery of new β-elemene derivatives is of increasing interest. We recently reported that the compound 13,14-bis(cis-3,5-dimethyl-1-piperazinyl)-β-elemene (IIi), a novel β-elemene derivative with a cis-2,6-dimethylpiperazine substitution, is a potent agent for inhibiting the proliferation of SGC-7901 and HeLa cells. In the present study, we further verified that IIi is cytotoxic to a wide spectrum of human cancer cells in culture, including those of breast, ovarian, lung, gastric, hepatocellular and colon cancer, as well as leukemia cell lines, with an average IC_50_ of 3.44 *μ*mol/l. Notably, IIi showed significant cytotoxicity in two multidrug-resistant (MDR) cell lines, with an average resistance factor (RF) of 1.66. Moreover, in mice with S-180 sarcoma xenografts, the intraperitoneal administration of IIi inhibited tumor growth. The immunoblotting study showed that treatment with IIi decreases phosphorylated p70S6K1 and 4EBP1 levels in the human breast cancer MCF-7 and MDA-MB-468 cells. In the MCF-7 cells, IIi also significantly increased the expression of cleaved LC3. This indicated that IIi inhibits mTOR activity and induces autophagy. The mTOR inhibitory function and the potent antitumor activity, taken together with the appreciable anti-multidrug resistance action, shows IIi to be a novel potential antitumor agent, which merits further research and development.

## Introduction

According to the World Health Organization (WHO) statistics, breast cancer is the most common type of cancer in females worldwide in developed and developing countries (1). It is estimated that 226,870 females will be diagnosed with breast cancer and 39,510 will have succumbed to the disease in the United States in 2012. According to the National Institute of Health (NIH), breast cancer is the second leading cause of cancer-related mortality, following lung cancer (2). Data from the Ministry of Health of The People’s Republic of China (MOHC) show that in China the incidence of breast cancer has been rising steadily in the last few years and that it is the most commonly diagnosed cancer in females. Elemene has been approved by the State Food and Drug Administration (SFDA) of China for the treatment of advanced cancers, including breast, gynecological and lung cancer. The essential oil extracted from *Curcuma wenyujin*, Y.H. Chen et C. Ling, a traditional Chinese herbal medicine, is a mixture containing β-, γ- and δ-elemene. β-elemene is the major active component (1).

**Figure 1:**
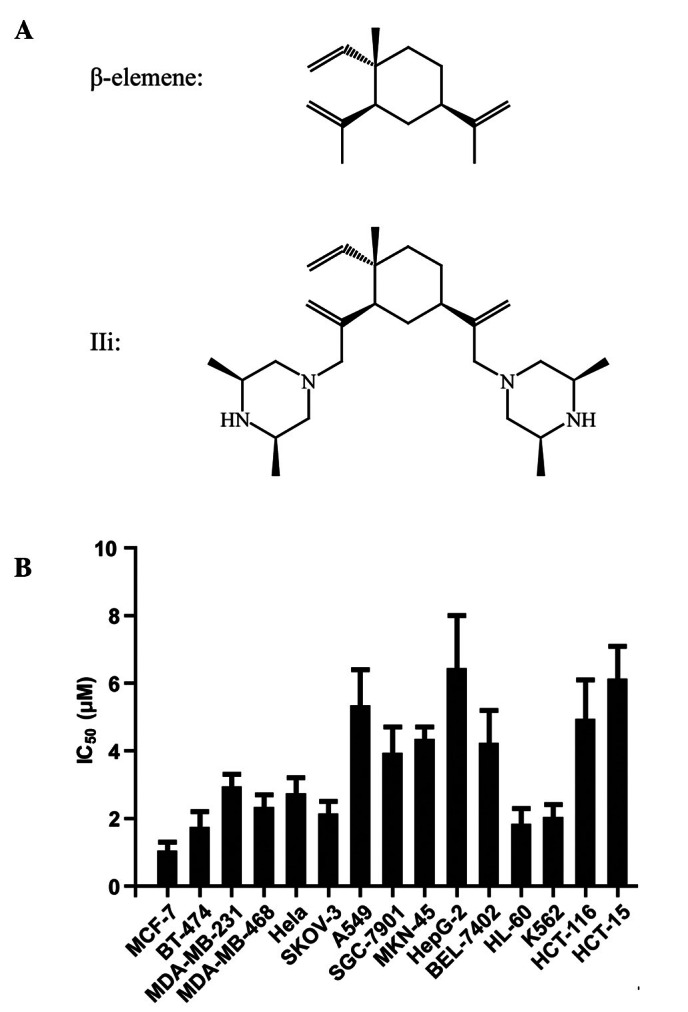
Antitumor activity of IIi *in vitro*. (A) Chemical structure of IIi and β-elemene. (B) Cytotoxicity of IIi against a panel of human tumor cell lines. Cells were treated with various concentrations of IIi for 72 h. Cell viability was determined by sulforhodamine B (SRB) assay or cell counting kit-8 (CCK-8). The columns show the mean IC_50_ values of three independent experiments and the bars represent the SD. IIi, 13,14-bis(cis-3,5-dimethyl-1-piperazinyl)-β-elemene.

Although β-elemene has been shown to inhibit tumor cell growth and an elemene emulsion has been approved for cancer treatment (3–7), the objective response rate with elemene has been modest in the majority of tumor types. Moreover, β-elemene is an essential oil with poor water-solubility and stability; therefore its use in cancer treatment is limited. To improve the activity and solubility of β-elemene, we synthesized a series of derivatives which contained piperazine, morpholine, tetrahydropyrrole, thiophenylethylamine or cyclohexamine groups in a previous study. Among these derivatives, 13,14-bis(cis-3,5-dimethyl-1-piperazinyl)-β-elemene (IIi) was found to be one of the most potent agents for inhibiting the proliferation of human cancer cells and it also demonstrated an inhibitory effect on mTOR (8) The structures of β-elemene and IIi are shown in Fig. 1A.

In the present study, we aimed to investigate the antitumor activities of IIi *in vitro* and *in vivo,* and to elucidate the possible associated mechanisms of action. The results show that IIi inhibited the proliferation of a wide spectrum of human cancer cells *in vitro*, including breast, ovarian and lung cancer cells. IIi also inhibited the growth of S-180 sarcoma in mice. The present study also showed that IIi inhibits mTOR activity and induces autophagy in breast cancer cells. These findings may aid in the verification of the antitumor activity of IIi and enable the rational design of a novel elemene analog.

## Materials and methods

### Cell culture and reagents

MCF-7, BT-474, MDA-MB-231, MDA-MB-468, HeLa, SKOV-3, A549, HepG-2, BEL-7402, HL-60, K562, HCT-116 and HCT-15 cells were obtained from the American Type Culture Collection (ATCC; Manassas, VA, USA) and maintained in appropriate medium, as suggested by the ATCC. MKN-45 and SGC-7901 human gastric cancer cells were obtained from the Cancer Research Foundation of Japan and were cultured in RPMI-1640 medium supplemented with 10% fetal bovine serum (FBS). The cells were incubated in a humidified atmosphere of 95% air with 5% CO_2_ at 37°C. β-elemene was obtained from Yuanda Pharmaceuticals (Dalian, China). IIi was obtained from the Key Laboratory of Structure-Based Drug Design and Discovery of Shenyang Pharmaceutical University, China. The IIi compund was synthesized using similar methods to those described in our previous study and characterized with infrared (IR) spectroscopy, proton nuclear magnetic resonance (^1^HNMR) spectroscopy, mass spectrometry and elemental analyses (6).

This study was approved by the Ethics Committee of Taizhou College, Taizhou, China.

### Immunoblotting and immunofluorescence

Immunoblotting and immunofluorescence analysis were conducted with standard procedures, using antibodies against S6K1, phosphorylated S6K1, 4EBP1, phosphorylated 4EBP1, LC3 (Cell Signaling Technology, Beverly, MA, USA) and GAPDH (Ding Guo Biotechnology, Beijing, China).

### Animals and antitumor activity in vivo

Seven-week-old specific pathogen-free male Kunming mice (weight, 18–22 g) were supplied by the Laboratory Animal Center of Shenyang Pharmaceutical University, Liaoning, China. The mice were inoculated subcutaneously into the right armpit with S-180 sarcoma cells. After 24 h, normal saline, cyclophosphamide (CTX), β-elemene and IIi were administered by intraperitoneal injection for 7 days. Following treatment, the animals were sacrificed by cervical dissociation and the solid tumors were removed and weighed. The inhibition rate was calculated as: [(Average tumor weight of normal saline group − average tumor weight of test group) / average tumor weight of normal saline group] ×100.

### Statistical analysis

Data are presented as the mean ± SD, and significance was assessed with the Student’s t-test. P<0.05 was considered to indicate a statistically significant difference.

## Results

### IIi inhibits cancer cell proliferation in vitro

As we had previously shown that IIi was able to inhibit the proliferation of HeLa and SGC-7901 cells during an experiment for drug screening (8), in the present study, we first verified the effects of IIi on the growth of human cancer cells in a wide spectrum of cell lines. The IC_50_ of IIi on the K562 and HL-60 cells was determined using a cell counting kit-8 (CCK-8; Beyotime Institute of Biotechnology, Haimen, China), while a sulforhodamine B (SRB) assay was used for the other cells. The cells treated with dimethylsulphoxide (DMSO) were used as the vehicle control. IIi displayed potent cytotoxicity in a dose-dependent manner in diversified cancer cell lines, including those of breast, ovarian, lung, gastric, hepatocellular and colon cancer, as well as leukemia cells (Fig. 1B). The average IC_50_ values of IIi against the 15 human tumor cell lines that were tested was 3.44 *μ*mol/l, and each IC_50_ was below 10 *μ*mol/l. The breast and ovarian cancer cells and the leukemia cells appeared to be more sensitive to IIi, with average IC_50_ values of 1.98, 2.40 and 1.90 *μ*mol/l, respectively.

### IIi overcomes drug resistance in vitro

To determine whether IIi was able to overcome drug resistance, the effect of IIi was examined on two multidrug-resistant (MDR) sublines, K562/A02 and MCF-7/Adriamycin. The drug-sensitive parental K562 and MCF-7 cell lines and conventional anticancer drugs Adriamycin and β-elemene were used as references. IIi and β-elemene displayed significant cytotoxicity in the MDR sublines examined, with average IC_50_ values of 2.7 and 318.2 *μ*mol/l, respectively, which were close to those values observed in the corresponding parental cell lines (average IC_50_, 1.7 and 281.8 *μ*mol/l). The average resistance factor (RF) of IIi on the MDR cells was 1.66 vs. 107.28 for Adriamycin (Table I).

### IIi arrests tumor growth in mice implanted with S-180 sarcoma

Kunming male mice inoculated with S-180 sarcoma cells were used to determine the effect of IIi on tumor growth *in vivo*. IIi showed dose-dependent effects on the tumor growth in mouse S-180 sarcoma models (Table II). Intraperitoneal administration of IIi at a dose of 40 mg/kg for 7 days reduced tumor growth by 50.4% and, at 20 mg/kg for 7 days, reduced tumor growth by 26.0%. CTX and β-elemene at doses of 30 and 50 mg/kg with the same schedule reduced tumor growth by 73 and 42.8%, respectively.

### IIi inhibits the mTOR target

**Figure 2:**
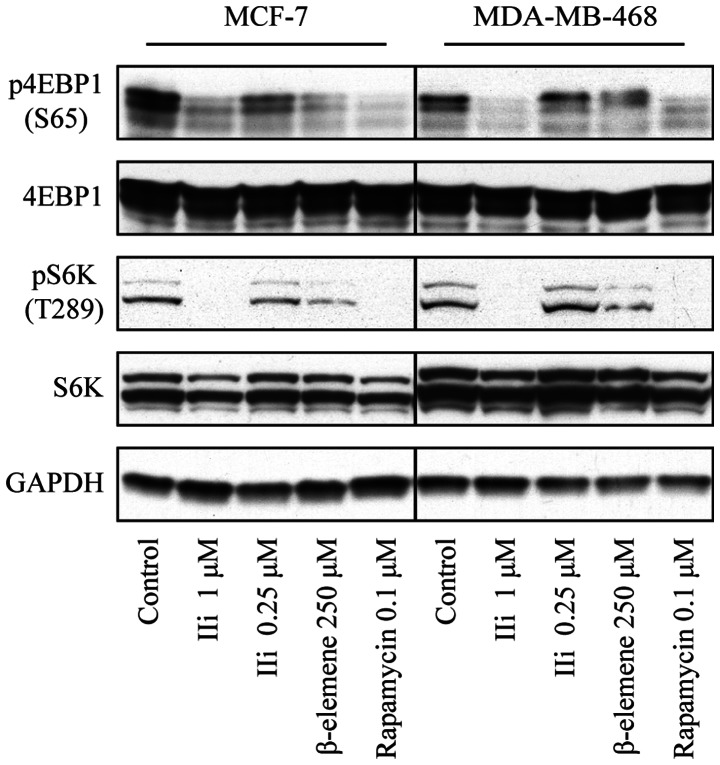
Effects of IIi on the mTOR pathway in breast cancer cells. The cells were treated with vehicle, IIi, β-elemene or rapamycin for 2 h and analyzed by immunoblotting. GAPDH was employed as a loading control. The data shown are representative of at least three independent experiments. IIi, 13,14-bis(cis-3,5-dimethyl-1-piperazinyl)-β-elemene.

In our previously published paper, we demonstrated that IIi could inhibit the mTOR pathway in K562 cells (6). In the present study, we explored whether IIi demonstrated the same function in breast cancer cells. The downstream effectors of the target protein were examined in MCF-7 and MDA-MB-468 cells upon IIi treatment. β-elemene and rapamycin were used as references. As shown in Fig. 2, IIi functioned as the mTOR inhibitor in a dose-dependent manner. IIi treatment at 0.25 *μ*mol/l for 2 h slightly decreased the phosphorylated p70S6K1 and 4EBP1 levels in the MCF-7 cells, but appeared to have no function in the MDA-MB-468 cells. However, the exposure of the MCF-7 and MDA-MB-468 cells to IIi at a dose of 1 *μ*mol/l significantly decreased the phosphorylated p70S6K1 and 4EBP1 in the two cells within 2 h, indicating that the mTOR activity was inhibited significantly by IIi. Rapamycin and β-elemene, at doses of 0.1 and 250 *μ*mol/l with the same schedule, showed a similar inhibitory effect.

### IIi induces autophagy in MCF-7 cells

**Figure 3:**
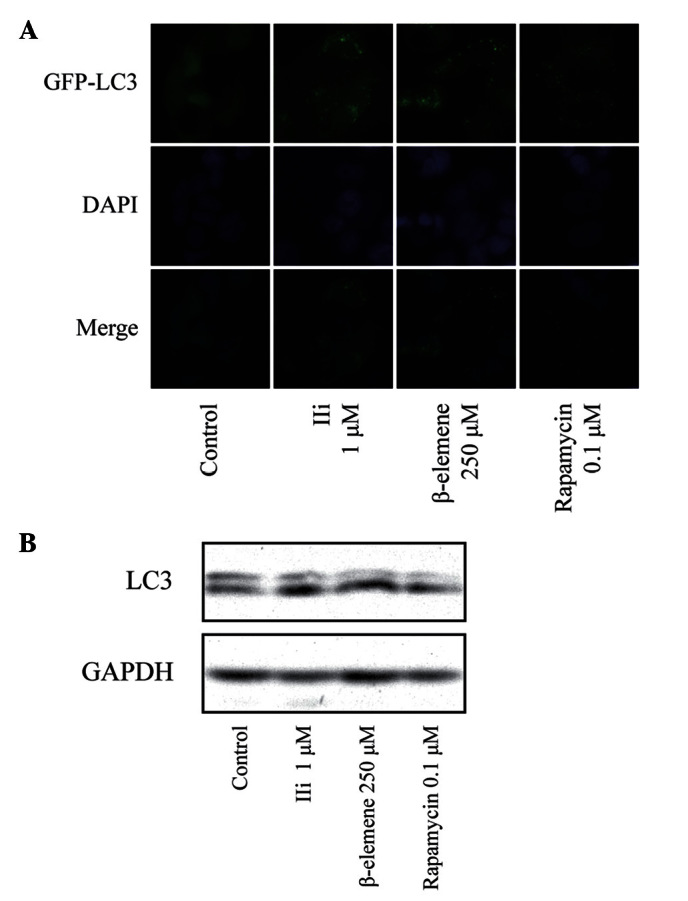
IIi induces autophagy in human breast cancer MCF-7 cells. (A) MCF-7 cells overexpressing EGFP-LC3 were treated with or without 1 *μ*mol/l IIi, 250 *μ*mol/l β-elemene and 0.1 *μ*mol/l rapamycin for 2 h. The cells were fixed with 4% paraformaldehyde (PFA)-PBS for 15 min at room temperature and then the fix solution was washed with PBS three times. The punctate pattern of the EGFP-LC3 fusion protein (green) was visualized with fluorescence microscopy at x40 magnification. Nuclei were stained with DAPI (blue). (B) MCF-7 cells were treated with or without 1 *μ*mol/l IIi, 250 *μ*mol/l β-elemene and 0.1 *μ*mol/l rapamycin for 2 h, and then harvested for protein analysis. Cell lysates were resolved in SDS-PAGE and probed with a specific antibody against LC3. GAPDH was employed as a loading control. Data shown are representative of at least three independent experiments. IIi, 13,14-bis(cis-3,5-dimethyl-1-piperazinyl)-β-elemene.

It has been reported that the mTOR inhibitors produce antitumor activities and induce autophagy (9), and that β-elemene also induces autophagy in certain cancer cells (10). As IIi has been confirmed as a novel β-elemene derivative with mTOR inhibiting activity, further examination of whether IIi was able to induce autophagy was performed in MCF-7 cells expressing LC3 fused to EGFP. As shown in Fig. 3A, a diffused distribution of LC3 was observed in the control cells, whereas a punctate pattern of LC3 was observed in the IIi-treated cells. To further confirm whether IIi was able to induce autophagy in the MCF-7 cells, an immunoblotting analysis was used to detect the cleavage of the LC3 protein. As shown in Fig. 3B, western blot analysis showed a significantly increased expression of cleaved LC3 in the IIi-treated MCF-7 cells. These results suggested that IIi induced autophagy in the MCF-7 cells.

## Discussion

In the present study, we showed that IIi, a novel β-elemene derivative, inhibits the proliferation of human cancer cells in a dose-dependent manner in numerous cancer cells, including breast, ovarian and lung cancer cells, and also inhibits the growth of S-180 sarcoma *in vivo*. IIi was also found to inhibit mTOR activity and induce autophagy in breast cancer cells. The results indicate that IIi may be a potent new anticancer drug candidate or leading compound.

The synthesis and evaluation of promising novel anticancer compounds remains an important challenge for new anti-cancer drug idenitifications. The rational optimization of lead compounds from natural products is one of the practical strategies for new drug development (11). Structural modifications have been demonstrated to successfully increase water solubility and/or antitumor activity of several natural compounds, including salvicine (12), camptothecin (13) and taxol (14). β-elemene as a novel anticancer herbal medicine has shown a range of antitumor effects *in vitro* and *in vivo*(4–7). It has been approved by the State Food and Drug Administration of China for the treatment of malignant effusions and certain solid tumors, following the positive results of phase III trials (7). However, β-elemene inhibits cell growth only at high concentrations and is an essential oil with poor water-solubility and stability. Previously, we synthesized and characterized a series of β-elemene derivatives and demonstrated that IIi is the most potent agent for inhibiting the proliferation of the human cancer cells HeLa and SGC-7901 (8). In the present study, we first used a variety of breast cancer (MCF-7, BT-474, MDA-MB-231 and MDA-MB-468), ovarian cancer (HeLa and SKOV-3), lung cancer (A549), gastric cancer (MKN-45 and SGC-7901), hepatocellular cancer (HepG-2 and BEL-7402), leukemia (K562 and HL-60) and colon cancer cell lines (HCT-15 and HCT-116) to evaluate the potential of IIi to induce cytotoxicity. The results show that IIi treatment significantly affected cell viability at lower concentrations, with IC_50_ <10 *μ*mol/l. Furthermore, the results show that the IIi treatment led to a significant reduction in the tumor size in mice implanted with S-180 sarcoma, when compared with untreated animals with tumors. Thus, the effectiveness of IIi in human cell lines and in mice makes it a potential cancer therapeutic agent. However, this requires further investigation in human cancer xenografts.

mTOR, a serine/threonine kinase, sits in the center of the PI3K pathway and has been validated as an important target for chemotherapy (15,16). The rapalogs, temsirolimus (Torisel and Wyeth) and everolimus (Afinitor and Novartis), were approved by the FDA for the treatment of cancer. However, the objective response rates with the rapalogs were modest in the majority of tumor types and highly variable (17,18). One important strategy to improve the efficacy of mTOR inhibitors is to identify new drugs over rapalogs (19). The present study showed that IIi decreases phosphorylated p70S6K1 and 4EBP1 in human breast cancer MCF-7 and MDA-MB-468 cells in a dose-dependent manner. It appears that IIi is a new mTOR inhibitor. Additional experiments are required to determine the possible mechanisms involved, including the interaction with FKBP-12 and the effects on the mTOR kinase domain. Among the signaling pathways implicated in the control of autophagy, the most well-characterized autophagy regulator to date is mTOR. Increased autophagic activity has been frequently observed in malignant cells in response to treatment with therapeutic mTOR inhibitors (9). In the present study, we also showed that IIi induces human cancer cell autophagy, in an identical manner to the mTOR inhibitor, rapamycin.

In summary, we have shown that IIi may be a potential cancer therapeutic agent candidate with mTOR inhibitory activity. However, additional animal models and clinical research are required to evaluate the effectiveness and safety of IIi.

## Figures and Tables

**Table I:** Cytotoxicity of IIi in MDR and drug-sensitive parental cell lines.

	IC_50_ (mean ± SD, n=3) (*μ*M)			IC_50_ (mean ± SD, n=3) (*μ*M)		
Compounds	K562	K562/A02	RF	MCF-7	MCF-7/ADR	RF
IIi	2.2±0.5	3.1±0.4	1.41	1.2±0.2	2.3±0.3	1.92
β-elemene	256.7±49.5	309.8±43.2	1.21	306.9±60.7	326.7±69.2	1.06
ADR	0.4±0.2	50.2±7.8	125.50	1.8±0.4	160.3±38.2	89.06

Resistance factor (RF) was calculated as the ratio of the IC_50_ value of the MDR cells to that of the corresponding sensitive parental cells. IIi, 13,14-bis(cis-3,5-dimethyl-1-piperazinyl)-β-elemene; ADR, Adriamycin; MDR, multidrug-resistant. P<0.05, vs. NS group

**Table II:** Effects of IIi on the growth of S-180 sarcoma in mice.

Treatment group	Dosage (i.p.)/(mg/kg/day) x no. of days	Mouse number (start/end)	Body weight (g)		Tumor weight (g)	Inhibition rate (%)
			Start	End		
NS	-	20/20	20.02±2.13	23.20±4.01	1.19±0.12	-
CTX	30×7	10/9	20.03±1.95	17.59±2.34^b^	0.31±0.09^a^	73.9
β-elemene	50×7	10/10	20.24±1.87	23.14±3.56	0.68±0.13^a^	42.8
IIi	40×7	10/9	20.64±1.94	19.62±3.47	0.59±0.14^a^	50.4
	20×7	10/9	20.00±1.83	19.64±2.43	0.88±0.23^b^	26.0
	10×7	10/10	20.11±1.96	22.83±1.64	1.06±0.14	10.9

Values are the mean ± SD of at least three independent experiments;

a P<0.01,

b P<0.05, vs. NS group. A Student’s t-test was used to assess the significance of the tumor growth inhibition of each group compared with the NS-treated group. NS, normal saline; CTX, cyclophosphamide; IIi, 13,14-bis(cis-3,5-dimethyl-1-piperazinyl)-β-elemene; i.p., intraperitoneal.
